# A Web-Based Intervention to Support a Growth Mindset and Well-Being in Unemployed Young Adults: Development Study

**DOI:** 10.2196/59158

**Published:** 2024-11-08

**Authors:** Ingjerd J Straand, Asbjørn Følstad, Burkhard C Wünsche

**Affiliations:** 1 Department of Social Work University of Stavanger Stavanger Norway; 2 Sustainable Communication Technologies Sintef Digital Oslo Norway; 3 Computer Science Department The University of Auckland Auckland New Zealand

**Keywords:** web-based intervention, positive psychology, mental health, user experience, persuasive design

## Abstract

**Background:**

Engaging young adults in the labor market is vital for economic growth and well-being. However, the path to employment often presents setbacks that impact motivation and psychological functioning. Research suggests exploring positive psychology interventions in job-seeking and scaling the delivery of these using technology. However, dropout rates are high for self-administered psychological interventions on digital platforms. This challenge needs to be addressed for such platforms to be effective conveyors of psychological interventions. This study addresses this challenge by exploring user-oriented methods and proposes persuasive features for the design and development of a new web-based intervention targeting young unemployed adults.

**Objective:**

This study aims to provide an overview of a new positive psychology wise intervention, including its theoretical underpinnings and human-centered design methodology, targeting young, unemployed adults.

**Methods:**

Researchers collaborated with designers, developers, and stakeholders to design a web-based positive psychology intervention that leverages evidence-based wise interventions. Key improvements and adaptations were explored through formative usability testing with 13 unemployed young adults aged between 18 and 25 years (the target population). Qualitative usability testing data were collected, analyzed, and integrated into the ongoing design process as iterative improvements.

**Results:**

The result of this study is a modular intervention web application named RØST, designed to align with the user needs and the preferences of the specific end-user group of unemployed young adults. During the project, this application evolved from early concept sketches and prototypes into a developed solution ready for further testing and use. Insights from both end-user feedback and rich user observation gained in the study were used to refine the content and the design. To increase targeted end users’ motivation, persuasive design features including praise, rewards, and reminders were added. The web application was designed primarily to be used on mobile phones using text messaging for reminders. The development process included technical and data protection considerations.

**Conclusions:**

This study offers valuable insights into developing psychological or behavioral interventions to support unemployed young adults by documenting the design process and the adaptation and combination of diverse theoretical and empirical foundations. Involving stakeholders and end users in the development enabled relatable content development and resolved potential usability problems. An essential implication is the finding that end-user feedback and insights are crucial in shaping interventions. However, we experienced tensions between the evidence-based interventions and the human-centered design approaches. These tensions were not resolved and highlighted a need for ongoing user motivation support through monetary rewards, which were incorporated into the final web app design.

## Introduction

### Background: Positive Psychology for Unemployed Young Adults

Web-based interventions are typically self-guided programs attempting to create positive change through an interactive presentation of health-related material [1]. Our study investigates the design and development of a web-based intervention relying on positive psychology [2] to achieve beneficial change in young unemployed adults. The specific objective of the intervention is to increase the users’ likelihood for engaging successfully with training, work, or employment. The successful engagement of young adults in the labor market plays a vital role in promoting personal well-being, driving economic growth, and fostering social cohesion [3]. Research suggests the importance of exploring further how to build psychological well-being and resilience [4,5] to cope with setbacks and challenges, which will occur as part of a job-seeking process [6-8]. The research idea of this study was thus that unemployed young adults could benefit from building confidence in the possibility to learn and improve and through normalizing and providing strategies for overcoming common challenges and stress, which occur in job-seeking process and in a young adult’s life in general.

### Wise Interventions

To provide young unemployed people with alternative interpretations of their situation and a mindset oriented toward learning and change, we draw upon the concept of *wise interventions*, as established in prior research [9]. Wise interventions are psychological interventions aimed at addressing social and personal challenges [10]. This field suggests that altering a maladaptive interpretation of a situation or an ability may change how the individual engages with their contexts in ways that lead to positive changes [11]. More specifically, our approach is guided by the previous research demonstrating that a time-limited and web-based wise intervention can improve stress management and enhance motivation and learning among young adults [12-14].

In the United States, the National Study of Learning Mindsets (NSLM) [13,15] developed and tested a web-based growth-mindset intervention. The NSLM intervention has evolved through years of iterative development originating from early paper-based interventions designed as newspaper articles distributed to students [16]. Over time, it has progressed toward web-based interventions (refer to the study by Blackwell et al [17]) and has also been translated and adapted to other institutional contexts (refer to the studies by Rege et al [12] and Dweck and Yeager [16]). A Norwegian adaptation, U-SAY, has been studied in experiments in a Norwegian educational context [12,18]. Both U-SAY and NSLM use reading and writing exercises to communicate a “growth mindset”—the belief that human capacities are not fixed but can be developed and increased in response to efforts, good strategies, and help from others [16] and a “stress-can-be-enhancing mindset” [14,19], which offers a more positive interpretation of psychological stress responses [19,20]. The content is carefully crafted to be relevant and meaningful to school students with clear and consistent messaging.

### The Challenge of Low User Motivation

The NSLM and U-SAY interventions are designed for use in controlled contexts such as classrooms [15,16], administered through PCs with headphones [21]. Thus, researchers can control when and how the intervention content is being consumed and make sure there are no distractions. Such contexts provide structures to piggyback on [16] to deliver the intervention and social incentives to comply with the use of the intervention (eg, established routine, doing what the teacher says, etc). Despite falling under the umbrella term of web-based interventions, the NSLM and U-SAY interventions represent structured textual content digitized and iterated from the original paper-based intervention. Both interventions consist of 2 short 30-minute reading lessons in combination with writing reflection tasks. The interventions are not designed to be particularly engaging as interactive experiences; they are rather static and linear but then the intervention is only competing with what would normally take place in the classroom [16].

By contrast, fully self-administered web-based interventions, lack these structures and suffer from low user adherence as a consequence [22]. In uncontrolled environments, it is up to 3 times more likely that young adults will drop out before the intervention has been completed [23]. This challenge is accentuated when the users are not interested in, or do not enjoy engaging with the intervention [24,25]. This underscores the importance of focusing on the interventions themselves to find a better fit with the preferences of young adults [26,27].

Use of design methods has been suggested as an approach to increase adherence and reach of web-based interventions [15,28]. In a human-centered design process, understanding the context of use and the needs of the user is a central part of the design work [29]. Design methods and participatory approaches which enable integration of user feedback into the designed outcome [30] have been shown to be helpful in enhancing psychological interventions [26]. In addition, Kelders et al [22] argue that the persuasive design features of the user experience (UX) in web-based interventions may predict higher adherence. In persuasive design, behavior is a product of motivation, ability, and prompts [31]. Transcending the “activation threshold” (ie, desired behavior) is possible by working to increase motivation (praise, rewards, fun), reducing barriers (making it easy, thereby lowering needed ability) and adding prompts (triggers) [31]. Thus, human-centered design methods and persuasive design features are central elements in the explorative work described in this development study.

### Objectives

This study aimed to design and develop a web-based intervention application using positive psychology to shape mindsets and engage young adults in the labor market. Due to the challenges posed by low motivation in the user population, we explored how to strengthen intervention usability and motivation through engaging with relevant user participants and other stakeholders. We were particularly interested in understanding how to leverage theoretical underpinnings and established empirical knowledge as part of the design process, while adapting this to the context of young unemployed adults. Our research question was thus: “How to design a self-administered wise intervention for young unemployed adults leveraging evidence-based, relevant wise interventions?” In this paper, we present the process and the resulting intervention—the RØST web app.

### Context

This study is part of a broader project, Career Learning App, which will use design methodology to adapt protocols from psychology into an application supporting young adults who are currently not in education, employment, or training. The new labor market requires workers with high competencies who are not afraid of change, challenges, and acquiring new skills. For people with weak beliefs in their capacity to learn, this could be a major risk factor for their exclusion from labor market. The aim of the research project is to design, develop, and test the effectiveness of a scalable, human-centered, and evidence-based growth-mindset intervention for young unemployed adults. The resulting web-based intervention will be used in large-scale randomized controlled trials (RCTs) in Norway [32]. The project will provide new knowledge about young adults who are not in education, employment, or training, and how the public welfare system may support them with psychological tools to improve resilience in entering the labor market or starting with education.

## Methods

### Study Design

The research question was explored through the design and development of a web app, intended as a self-administered wise intervention for young unemployed adults. The study used a human-centered design methodology [29,33] to ensure that the intervention would be relevant, user-friendly, and motivating to its users. Empathy with the end-user perspective may be particularly relevant in this context where motivation may be low in general, as indicated by the target group’s incomplete schooling [34] and frequent dropping in and out of employment and training [35].

**Figure 1:**
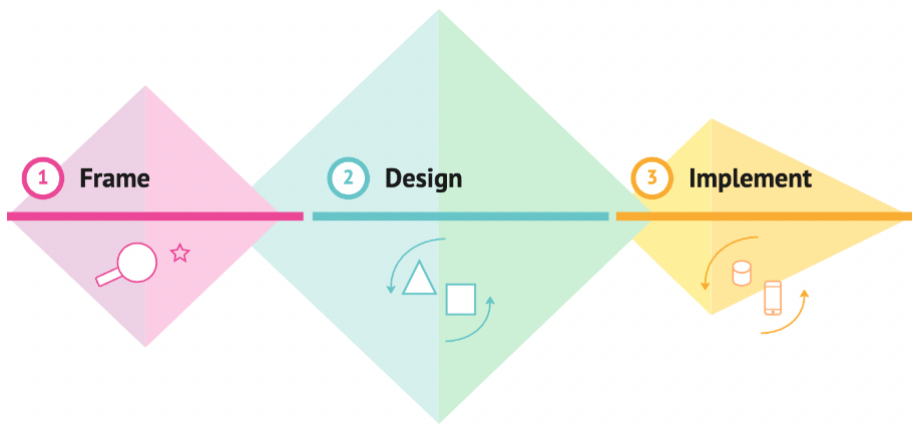
The design and development process of the intervention application visualized as a 3-step process.

In this process, researchers collaborated and engaged with designers, developers, and stakeholders, including potential end users, from October 2020 until the completion of the RØST application in December 2022. The process occurred in stages following a 3-step iterative design process, as outlined in Figure 1, consisting of both divergent (creative and intuitive) and convergent (logical and analytical) thinking processes [36-38].

The human-centered design process is a broad approach and may include many possible research methods to empathize with the user perspective. Specifically, this study used formative and qualitative usability testing [39] as a core method to evaluate concepts and prototypes and explore end users’ experiences. These usability testing sessions were conducted with the relevant end-user participants over Zoom (Zoom Video Communication, Inc; due to COVID-19 pandemic restrictions) and included a walk-through of the prototypes where users could comment freely what they were thinking about each screen. The sessions lasted 45 to 90 minutes and included semistructured and open interview questions.

The target of the usability sessions was to receive feedback on different prototype designs and ideas or inspiration for improvement. Thus, we tested prototypes of both high and low fidelity to capture participants’ opinions, reasoning, and attitudes regarding an interactive experience in relation to their own situation as unemployed. Participants were asked open questions such as “What do you think about what you see here?” and “Would this be helpful to you—or not?” Thus, the prototypes enabled exploration and functioned as boundary objects [40,41] that *framed* the conversations with end users.

### Data Analysis: Insights From Synthesis

Results from the usability testing were analyzed through the design process. Members of the core team took on the roles of observer and a moderator in the sessions with the participants. The observer wrote notes that could be used as input to a posttest summary of observations and findings, which represented key findings, highlights, and particularly interesting observations. These were established through a brief discussion between the moderator and the observer, combining the outcome of the discussion with that of visual diagramming in the form of affinity maps (the Jiro Kawakita method; refer to the study by Scupin [42]) and Post-it or notes of interesting quotes from the participants. The findings were shared either orally as a form of usability testing “debrief” in the weekly stand-up meeting or in writing on Slack (Slack Technologies, LLC). The feedback collected was then discussed in the core team and used to improve and iterate the design and content through new sketches and changes to prototypes [43]. This way, sensemaking of the collected data occurred through synthesis [44,45] where findings emerged through intuitive, holistic interpretations [46] and were applied to action through creative problem-solving to iterate on the designs.

### Involving Stakeholders and End Users

In total, 13 young adults (n=7, 54% men and n=6, 46% women) from the target group participated in the study, giving their feedback on the prototypes. In addition to the end users, a multitude of other stakeholders were also involved in some manner, either in cocreating activity to bring forth ideas or contributing to problem-solving, or they were consulted for their input and expertise for the intervention app design, content, or development.

A core team of 3 researchers (including author IJS), 2 UX designers, and 2 developers worked to bring forth the design and the build of the web app. The core team met at least once a week, but usually, at least some of the team members met daily or several times per week and collaborated closely on all aspects of the web app.

End-user participants represented the most central stakeholder to this study and were involved through taking part in usability testing, and gave feedback on the latest prototype designs. Beyond end-user participants, other stakeholders were also involved, consulted, or informed for quality check and expertise. This included other researchers with expertise in fields such as behavioral and wise interventions, psychology, and design methods. These researchers were involved approximately once per month and took part in workshops or were consulted as advisers, often about the contents of the app, via emails or meetings. This advisory group of researchers shared expertise from their respective fields while being outside of the ongoing and day-to-day designing process of the intervention app.

The remaining stakeholders were people that the core team engaged with on an ad hoc basis, either through meetings or emails to get their input or expertise. Some contributed by performing parts of the work, for example, editing or proofreading (communication staff). Others contributed via their knowledge of the user group, such as the caseworkers at the Norwegian Labour and Welfare Administration (NAV) who provided the core team with their experience-based insight into the user group specific to the local context. Furthermore, the caseworkers shared their thoughts on how they believed the user group would react to the different parts of the web app, particularly regarding the wording. Thus, the core team could adjust the prototypes before usability testing with participants. The case workers and the advisory researchers also contributed by recruiting end-user participants.

Finally, some of the stakeholders were important to liaise with as potential “gatekeepers” to the final web app because of their roles. This included data protection (data protection officers and IT and legal advisers at NAV and at the University), technical architecture (university IT staff), and content owners that could grant permissions to use third-party intellectual property for the intervention, including the use of design systems, photos and news media content.

### Recruiting

Inclusion criteria for the study were as follows: participants aged between 18 to 25 years and living in Norway or speaking Norwegian (due to in-app language) with experience of being unemployed or dropping out of school. Recruitment was done through partner stakeholders who deal directly with this population. The COVID-19 pandemic made in-person meetings impossible and also made it difficult to reach participants, for instance with the loss of physical premises to distribute flyers and so forth. Thus, caseworkers at NAV sent out invitations to take part in the study through their secure dialogue platform, and users who were interested could express their interest through a web-based form. This way, we recruited 8 young adults, which was a little below our aim. To avoid delays in the ongoing design process, we recruited additional 2 participants through a local individual placement and support program and 3 participants through snowballing. All testing sessions were done on the web via the videoconferencing platform Zoom (the lessons learned from conducting this research remotely are presented in the study by Straand et al [47]). The participants had diverse backgrounds and stated diverse reasons for their current situation, including unfinished schooling, mental illnesses, waiting for support from, for instance, NAV, and lack of training placements following vocational schooling.

Other stakeholders involved in this study were not recruited as research participants but rather contacted directly due to their specific roles or expertise. Some of them also had a defined role in the Career Learning App project.

### Ethical Considerations

The study was evaluated and approved by the Norwegian Centre for Research Data (approval number 131074). All end users provided explicit and written consent to participate in the study, and were given the opportunity to opt out at any time. Identifying information is omitted from all publications. End users were rewarded with gift cards of US $30 per session for their participation. Stakeholders were not rewarded for their participation.

## Results

### App Design and Development

#### Overview

**Figure 2:**
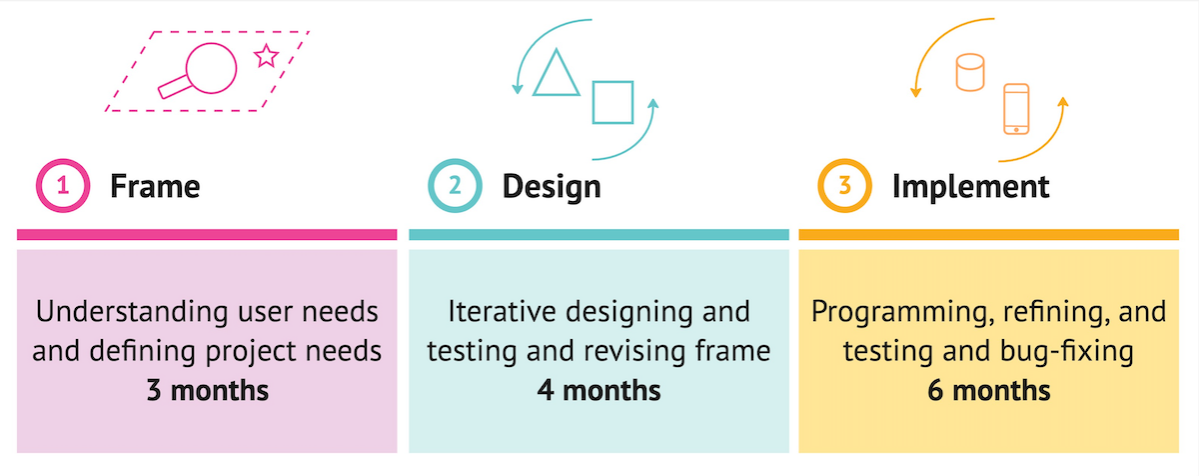
The time frame and the stages of the design process of the web application.

In this study, our aim was to both rethink the intervention’s content for it to be meaningful and relatable to the particular user group [16], and to develop an intervention technology that would support self-administered use on any device which also considered users’ potential lack of motivation. Therefore, we started from existing web-based growth-mindset interventions and applied a process that was iterative and agile to support learning through experimentation [15] and through frequent feedback from end-user participants and other stakeholders. The resulting RØST web app was therefore developed and designed in 3 main steps, with Figure 2 providing details on the time frame of the project.

The process was not strictly linear and involved moving back and forth between the different steps. An outline of the 3 steps is given in the subsequent sections.

#### Frame

Framing in design research and practice is an acquired expertise to reach a focused problem definition through critical questioning and interpretation of the problem space [48,49]. This research was initially scoped by the need to deliver a web-based intervention building on existing evidence-based interventions, specifically those of NSLM and U-SAY. Thus, framing in this study was our interpretation and understanding of the problem, existing data, and knowledge within that scope. Therefore, the core project team, which at this stage consisted of 3 researchers at the University of Stavanger with expertise in growth mindset, behavioral intervention design, and human-centered design (author IS), conducted a brief evaluation of the U-SAY intervention with 4 end-user participants to get a richer understanding of the user’s perspective and context.

The general sentiment toward U-SAY with these participants was a perception of the messaging to be positive and empathic in *tone*, but all 4 participants viewed the intervention to be more appropriate for a younger age group and that it was partially irrelevant (due to the school context), static, and repetitive. It also appeared “dated” in terms of visual design, and it was perceived to be overly reliant on reading textual material. There was a marked user preference for a mobile app or mobile design by all 4 participants. The research team also interpreted that the participants would prefer to have more control of the UX and flow, as they all expressed annoyance or dislike of the lack of such a possibility in U-SAY (eg, not being able to turn off the app audio or lack of freedom to explore and lack of interactivity). The users appreciated reflection questions that were included in U-SAY but were uncertain as to whether they would have answered these in the context of actual use. The questions were perceived to be difficult to answer, and the participants were unsure of what they would “get in return.” We also showed participants a publicly available resource for fostering a growth mindset (PERTS Mindset Kit [50]) to exemplify material designed for a more mature population (mentors, parents, and teachers). The Mindset Kit was regarded as much more professional, serious, and relevant by all participants.

The research team also gathered background information about the target end-user group to empathize with their challenges and needs, based on published research and conversations with caseworkers at NAV. This was not intended as a literature review but rather to provide rapid but rich background information to inspire creative problem-solving. A key insight was the substantial heterogeneity in the target group (refer to studies by the Organisation for Economic Co-operation and Development [3], Fyhn et al [51], and Mascherini [52]). Key commonalities within the target group were broad and encompassed educational and health issues, for instance. Many of the young adults who are outside of education and work in Norway have not completed high school [53]. Furthermore, they have mental health problems more often than other young adults [51,54].

The user-centered evaluation of current intervention designs along with collected background information and research experience from a gaming-based intervention for a similar target group [27] helped provide a direction for the forthcoming design work. The beginning is never really the beginning of participatory action–based research [55]: the research team had previous and recent experience evaluating a gaming-based intervention app with the same user group (refer to the studies by Straand et al [27] and Straand et al [47]) and could interpret findings from this limited set of participants against a plethora of past experiences and lessons learned. For instance, the need for supporting mobile use was, to some degree, established from a related study [27] and thus, even with such a limited number of participants, the research team felt confident in redirecting the design and development process to a mobile phone app or a web application designed for mobile use. All this collated material and past experience led to the construction of “conceptual hypotheses” in the form of 5 key insights that were put forward toward the end of the *framing* step (Textbox 1).

**Five key insights about the user group and their needs were put forward to guide and inspire forthcoming design exploration.**

**Key insights**

1. The target group consists of young adults, not teenagers. They want to be taken seriously with an intervention design reflecting the maturity of the users.
2. For the intervention to be perceived as relevant, intervention content about growth mindset should be closely linked to the situation of the users and how they can use the knowledge for change [56].
3. The target group is heterogeneous. Hence, it may be relevant to enable adapting the intervention (personalize it) to specific challenges and perspectives.
4. It is imperative that the intervention app has a very low threshold for use, enabling users to get started quickly and easily. We foresee that motivation for use may be low.
5. Due to potential limitations in basic education, reading and writing exercises may be challenging or frustrating for the target group.

These statements were intended to guide initial design exploration. On the back of the framing stage, we wrote a simple project brief for the needed design work using standardized university procurement documents and processes and asked 7 design agencies for proposals. In total, 4 proposals were received, and we picked a team based on a weighted evaluation of experience, availability, and cost.

#### Design

Upon entering the design stage, the core team was expanded to include 2 UX designers and a senior developer as consultants. The specification documents included a milestone plan which outlined a 3-month design process, starting with an opening workshop to share all relevant project information, including the existing interventions and findings from the usability testing and the conceptual hypothesis. Due to the COVID-19 pandemic, the core team could not meet up to work together but collaborated intensively using tools such as Figma (Figma, Inc), Zoom, Miro (RealtimeBoard, Inc), Slack, and Google Drive (Google LLC) to work together, meeting in web-based workshops, weekly stand-up meetings, testing and feedback sessions with end users, and group chats.

Design exploration involved investigating appropriate content, user interface (UI) appeal, and functionality needs. We mapped out existing mental health apps available on the web and in Google and Apple app stores (Apple Inc) and investigated ways to make the intervention more interactive and engaging. On the basis of exploring other self-administered intervention studies [23,25,26], it was established that adherence to the intervention would likely be a challenge. However, our current project framing and available resources would not allow a full exploration of highly engaging UXs, including advanced gamification mechanics. Ideas such as interactive games and the use of artificial intelligence and chatbots had to be dropped, although the research team felt that in particular, the exploring of conversational artificial intelligence could be a promising direction in a future study, as it may cater to a personalized and adaptive UX supporting features such as “Socratic questioning” to bring to light users’ existing interpretation of their situation to start questioning that interpretation [57].

Furthermore, we reviewed the effectiveness of different gamification mechanisms in similar applications. Li et al [58] performed a meta-analysis of gamification in educational settings and found that gamification was, in general, effective but effect size depended on user type (eg, more effective for primary school students than secondary school students), application area (eg, more effective for science than business and social science), and duration (eg, more effective for longer interventions). Krath et al [59] reviewed the theoretical basis of gamification and reported that the most frequently cited concept is self-determination theory. Gamification mechanics can fulfill users’ basic needs for autonomy, competence, and relatedness, for example, by providing users with badges that indicate skills and group membership (eg, “Expert”). The authors further found that flow theory was the second-most cited underlying mechanism making gamification effective. This means the key mechanisms making gamification effective are related to intrinsic motivation (eg, enjoyment and autonomy). Xiao and Hew [60] compared virtual with tangible rewards in an education setting and found that tangible rewards (sample answers and more practice questions) resulted in higher intrinsic motivation, behavioral engagement, cognitive engagement, and learning performance in the final examination. Thus, we incorporated prompts, praise, and rewards into the app to motivate users to adherence.

In accordance with the combined user feedback, research priorities, and budget, a decision was made to build a web-based application (ie, an application used in a web browser) with a responsive design suitable for all devices but with a primary focus on mobile use following a *mobile-first* best practice [61,62]. This meant that the users could choose the device for themselves. However, the mobile design was prioritized, and it was the mobile design prototypes that were presented to the participants in usability testing. There were no reactions or comments from end users that led us to question this decision.

We had been granted permission to use an existing “design system” from the NAV called Aksel [63]. This included a UI kit, an illustration library, and icons as Figma-files and a GitHub component library. Using a well-defined design system was a method to not only save resources but also to meet the highest standards in terms of accessibility, usability, and a professional-looking UI. However, the use of the NAV design system was a decision made with some doubts. Our previous research [27], along with media reports in Norway, indicated that mention of the NAV could elicit negative reactions. Specifically, young adults navigating the welfare system told us that they could get uneasy, worried, or “sick in their stomach” when they receive a message from the NAV. We reasoned that this had to do more with the perception of NAV as a gatekeeper of welfare rather than a perception of the UI.

Focusing on a simpler web app concept designed for mobile use and taking advantage of the NAV design system, we conducted usability testing on 3 major prototype revisions made in Figma with users from the target population who informed the ongoing design work. Users in the usability testing commented that they *liked* the design and had no negative feedback on the UI. It is our interpretation that they did not recognize the resemblance to the NAV UI. Further findings from usability testing and the implications for design are presented in the Insight From the Human-Centered Design Process section of this paper.

#### Implement

In this step, we explored options for technical development and solution architecture. Cost and benefits had to be considered, along with privacy, security, and data management. After initial considerations, the core team worked with 2 developers to engineer and build the solution iteratively. The development process also included considering data storage, authentication protocols, and technical aspects related to a forthcoming RCT to evaluate the effectiveness of the app. Thus, we had to redefine the architecture before the final version of the app could be completed, as more specialists in ethics, data security, and experimental research and legal advisers got involved. The content was also iterated on several occasions with help from copy editors and university researchers and then recorded by professional actors. In addition, there were several rounds of testing to weed out bugs and problems. The testers were mostly university staff, friends, and family but also included some end users. At this stage, feedback was used mostly to correct technical problems and minor usability issues.

The final app will be described in further detail in subsequent sections, but first we summarize some of the key findings from across usability tests in this study as overall insights.

### Insight From the Human-Centered Design Process

Throughout the process of design and development, involving end users as participants in usability testing influenced content revisions, helped tackle usability issues, and supported fine-tuning of strategies to increase user motivation. While the design and functionality of the web app is the central outcome of this study and is elaborated on in detail in the next section, it is important to also highlight some of the key findings that were drawn from the usability testing from across stages in the process and their implications for the ongoing design. The findings represent the interpreted sentiment and mood of the users, that is, what the users expressed directly or could be interpreted from their more indirect comments, gestures, or behavior.

**Figure 3:**
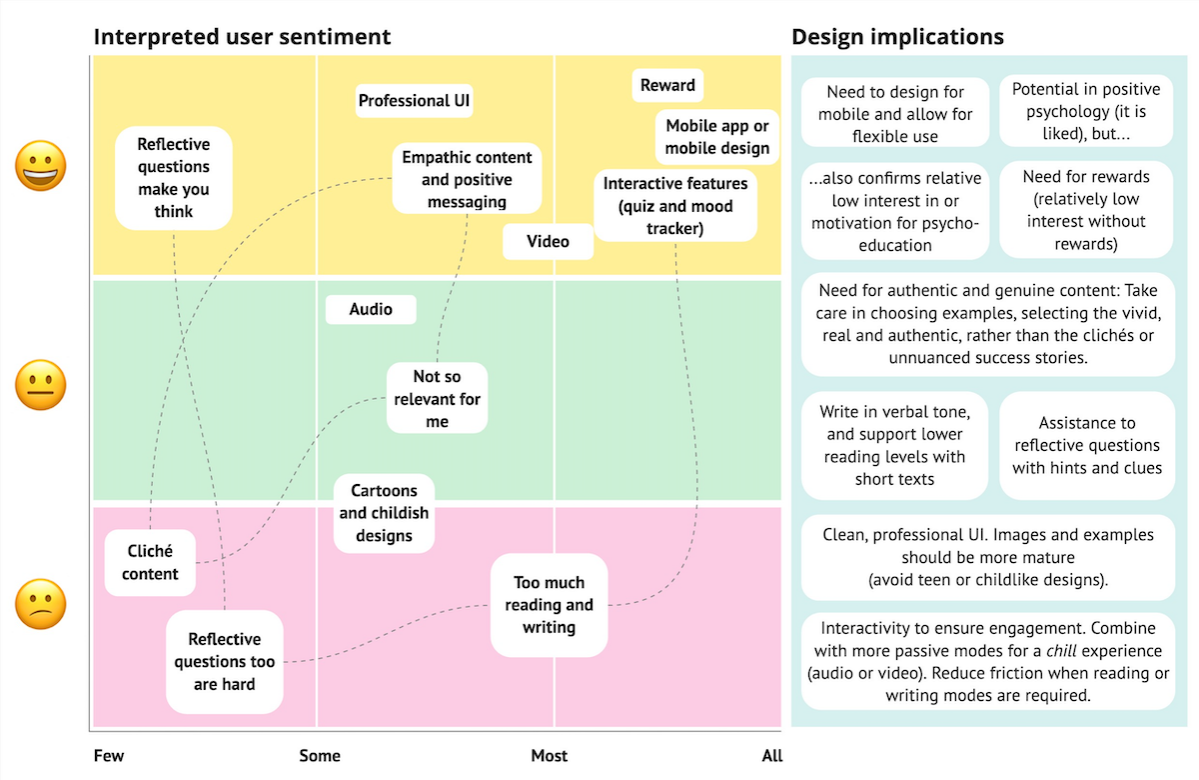
Opinions of the involved user participants and the interpreted implications for the ongoing design and content. UI: user interface.

For transparency on the prevalence of reported findings across participants and prototypes, we apply the following use of wording to describe the strength of each finding: All and nearly all (observed for the vast majority of relevant stakeholders and participants), most (observed for the majority of stakeholders), some (observed for a substantial minority of stakeholders), a few (observed for a small minority of stakeholders). These findings are summarized in Textbox 2 and visualized in Figure 3 with the derived design implications.

**Sentiments of the user participants involved in the study and the strength of the findings based on the number of participants who expressed similar views.**

**All and nearly all participants**

- Mobile use: All said the app should be designed for mobile use, for instance, to allow for flexibility in use and the phone is always available.
- Rewards motivate: The main reason for completing the app for nearly all participants would be to get a reward. Most did not see a direct relevance to their personal situation at this time. A few said they would complete it with no reward if they were required to do this by the Norwegian Labour and Welfare Administration, for instance.
- Professional: Nearly all felt the user interface should be “professional” in look and feel, whereas teenager-like interfaces or use of cartoons were seen as too childish.
- Interactive elements=more engaging and fun: The more interactive parts, such as quizzes and mood tracker were well-liked and appeared to be more engaging by nearly all.

**Most participants**

- Video content was perceived by most as positive and entertaining.
- Positive and empathic content: Most liked the positive messaging and empathic tone of voice underlying positive psychology, such as growth mindset. Some wished they had learnt about this at a younger age (“when they needed it most”). Most appreciated relatable content, including content with examples of people in similar situations or interests as themselves (but what was seen as relatable varied across participants).

**Some participants**

- Reading and writing: some expressed negative sentiments toward reading and writing.
- Audio: some liked the audio feature, describing it as “chill.”

**A few participants**

- Reflective questions: A few expressed a dislike for and showed indications of struggles with answering reflective questions. A few commented on how reflective questions made you think.
- Authenticity: A few expressed explicit dislikes to what was perceived as “cliché statements” and inauthentic examples.

### The RØST App

#### Overview

**Figure 4:**
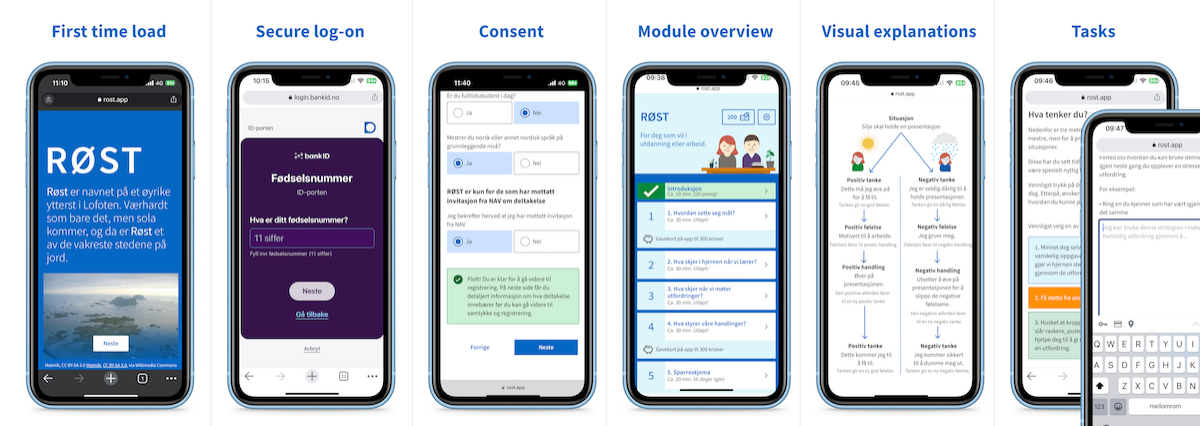
Overview of the RØST application and its features showing different screenshots from various parts of the application.

The main result of this project is the development of a web-based intervention application: RØST. RØST is built as a web app, to run in a browser using web technologies, including React front-end, Nest.js back-end, and the Sanity Content Management System. We used a third-party secure authentication, ID-porten, a log-in solution for web-based public and banking services in Norway (Figure 4).

As the user enters the solution for the first time, there is a short prequalification survey where the user has to enter a participant code, followed by electronic consent and user authentication. Then, the user is redirected to a survey tool (Qualtrics; Qualtrics International Inc) for the baseline measure surveys required for forthcoming studies. When the users are being redirected from the app solution into Qualtrics, they are automatically anonymized. We create a user ID consisting of a numerical string which is the only information shared with the Qualtrics servers. This allows us to separate personally identifiable information from the survey data and use data. After survey completion, the user is redirected and deanonymized back into the app. App use data and user inputs are stored on General Data Protection Regulation–compliant servers within Norway.

RØST is designed as a modular course with 6 sessions spread over 10 weeks. The strict pacing of the app allows for control of the UX, which is not only important for forthcoming experimental studies but is also beneficial from a learning perspective. The user needs time to learn and to let the messages “sink in,” and the content in a single session seemed to be “enough” for 1 week for most participants, evidenced by faster “clicking-through” behaviors after 1 module had been completed. However, testing revealed that this might not align with users’ preferences. Despite this, we recognize that there is a balance to be struck, as some level of structure may be necessary for the intervention and the control of the forthcoming RCT. In subsequent sections, we present the RØST content and selected important features, which are grounded in the human-centered design process and in past evidence-based interventions. Refer also to Multimedia Appendix 1 for further details.

#### Conceptual Grounding in Existing Evidence-Based Interventions

Unemployed young adults and young adults not in education or training are overrepresented in terms of mental health problems [53] such as depression [64], anxiety, and avoidance behaviors [65]. To develop the web app RØST, targeting the challenges faced by those among the young adult population who are unemployed or not in education, we drew upon the material from various intervention sources. In this section, we present the contents of the RØST application and its theoretical and empirical underpinnings.

The initial content framework was inspired by the material from the Norwegian-adopted web-based growth-mindset intervention U-SAY [18], supplemented with content from a synergetic growth mindset and a stress mindset intervention, providing strategies for managing stressful events and fostering a more constructive interpretation of stressors [14]. In addition, we integrated techniques from cognitive behavioral therapy to effectively address negative emotions [57] and Mental Contrasting Intention Intervention (MCII) to promote goal-setting activities [66-71] (more information on MCII is in the Setting Goals section).

The overarching approach to adapting these interventions involved retaining core content and principles but adjusting the examples used to suit the context of job seeking. In addition, we expanded the intervention and made it more relatable by incorporating real-world examples from news media. We have also shortened the text significantly to meet the user’s desire for less reading and repeated core messages in a video. We have provided an example in Textbox 3 on how the context was adapted from a school setting to a work-life setting.

Furthermore, the web app content uses various strategies and principles that are common among wise interventions [10,11] to communicate the messages and persuade the intended audience, as listed in Textbox 4. These are captured from the literature connected with the wise interventions (refer to the study by Walton and Crum [11]).

**Figure 5:**
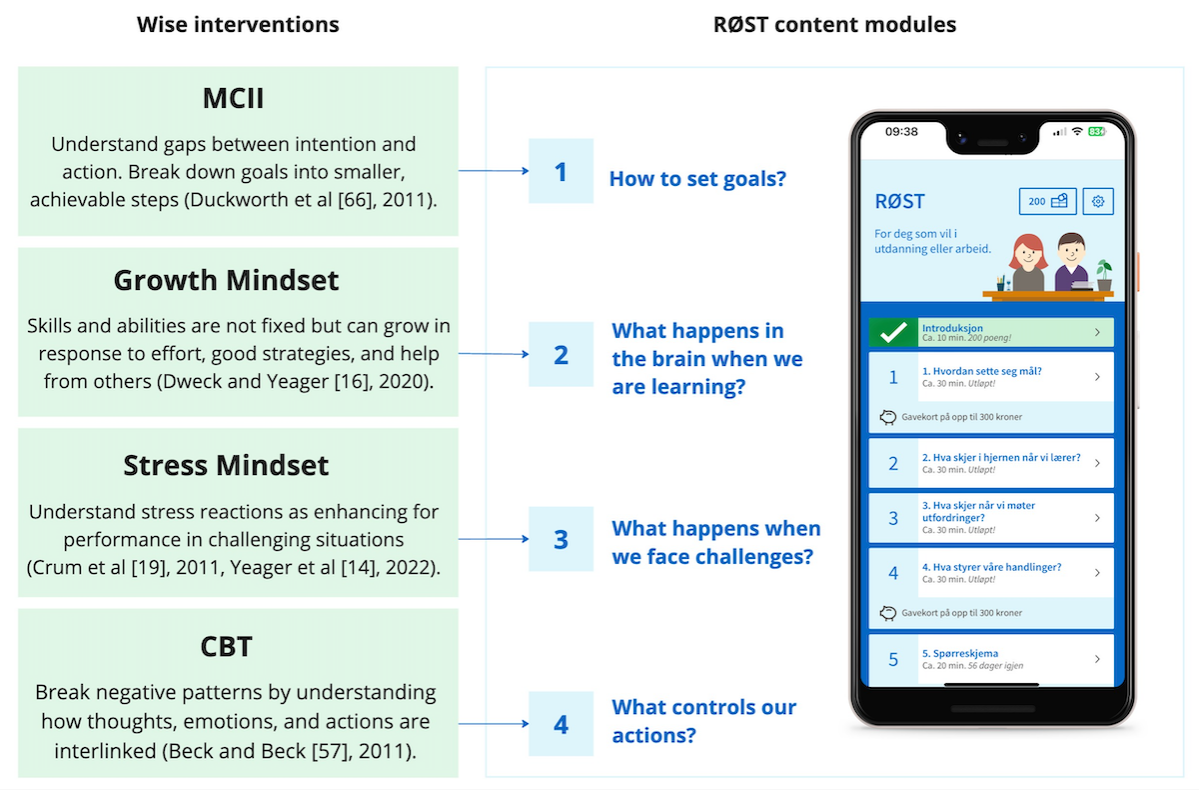
The content of the app is grounded in a range of previous interventions tailored to meet the needs of the target population of young unemployed adults [14,16,19,57,66]. CBT: cognitive behavioral therapy; MCII: Mental Contrasting Intention Intervention.

Figure 5 presents how the different chapters of RØST correspond with different past and evidence-based wise interventions. The growth-mindset intervention (in the introduction and chapter 2), teaches students about research in neuroscience, showcasing the brain’s capacity for growth. It conveys the understanding that challenging learning experiences as well as navigating through confusion can foster the formation of new neural connections. It communicates this message by using the metaphor that you can “train your brain as a muscle” by engaging yourself with tasks that are challenging [12,16,76] and then, through explaining in detail, how skills and intelligence can be developed with effort, time, good strategies, and help from others.

**Examples of how context was adapted from the original intervention designed for classroom use in school to a new context of self-administered use in a web app targeting a more mature population group. The examples also demonstrate how content was shortened.**

**Original intervention**

- Name one or two demanding assignments at school that you really had to think hard and put in the effort to solve. You can, for example, take your math or Norwegian lessons as a starting point.
- Write your answer in a text document to be sent to the teacher after the program is finished. [from U-SAY] [18] [translated to English by authors]
- “People always tell me to relax and not stress about tests or big projects. But that never felt like good advice because I actually care a lot about what I’m doing and I feel motivated to do a good job.” [JH, a fourth year student]
- “I am glad someone finally took the time to explain that it’s a good thing that I’m not taking the easy road. It makes sense that telling yourself ‘don’t stress’ doesn’t work when you’ve chosen to do something hard but important.” [JH, a fourth year student]
- “Instead, I can use my body’s stress response to do better at the things that matter a lot to me. I feel way more comfortable knowing that my body and brain are working to help me even when I don’t know it—and that I can harness that power when I need to.” [JH, a fourth year student] [From Synergistic Mindset Intervention] [14]

**RØST web app**

- “Can you name a challenging task that you really had to think hard about to solve?”
- <Text input field> [From Røst, translated to English by authors]
- “People told me to relax and not stress before a job interview. But this didn’t feel like good advice, because I wanted to do well in the interview.” [JH job applicant]
- “I was happy to learn that taking the easy way out doesn’t help. When we are working towards something that is difficult and important to us, it does not help to just tell ourselves not to stress.” [JH job applicant]
- “I experience the stress responses better now that I know that my body and brain are working hard to help me—completely without me realizing it, and that I can use this when I need it.” [JH job applicant] [from Røst, Translated to English by authors]

**Key strategies used in the RØST application.**

**Key strategies**

1. Introduce a *sticky metaphor* for a growth mindset (“the brain is like a muscle”) [17]
2. Leverage *social incentives* by stating what other young adults have said about the program [72]
3. Present *relatable examples* to capture the user's attention and make it memorable [73]
4. Provide early summaries of the intervention content to *set user expectations*
5. Talk with, not to, users. Young adults would rather *conform to peers* [74] (avoid patronizing)
6. Communicate *lived and real experiences*, as these may be more impactful than theoretical constructs. Theoretical constructs and explanations can come after. Focus on *utility value* when introducing constructs [56]
7. Use constructive activities to support learning and *self-persuasion* by applying a “saying is believing” tactic [75]
8. Apply *paced learning and repetition of key information in various formats* to allow messages to “sink in”

In the stress mindset intervention (chapter 3) participants are introduced to the concept that stress can be beneficial (contrary to popular belief). It emphasizes an understanding of our psychophysiological stress response, characterized by symptoms such as sweaty palms, a racing heart, deeper breathing, and feelings of anxiety. These physiological changes are depicted as beneficial, as they energize the body and supply oxygenated blood to the brain and tissues, thus improving performance. The intervention conveys this message by presenting stress responses as strengths, making one ready to take on challenges [14]. Thus, both stress mindset and growth mindset are attempts to change users’ thought processes and actions through attributional retraining [77]. The content in the various modules is presented in further detail in Multimedia Appendix 1.

In the following sections, we describe specific features of the app that we have developed to address the absence of a structured learning environment and ensure participant engagement. These include (1) mood charting, (2) setting goals, (3) audio and video, and (4) persuasive design features: triggers, rewards, and praise. We expect that these features are essential for maintaining participant involvement, particularly in environments lacking the traditional structures found in educational settings

#### Mood Charting

**Figure 6:**
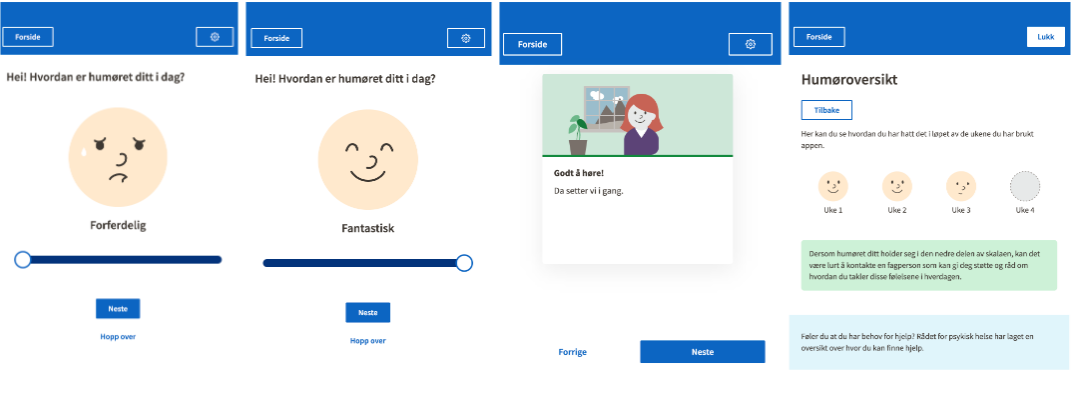
Mood charting functionality in the app with a possibility to track the user’s moods before each session. The mood slider can be moved by the user to indicate their current mood from “terrible” to “fantastic.”

Mood charting or mood tracking involves tracking the user’s mood at regular intervals and may be done using mobile phones [78]. In our app, we decided to include mood charting as an indication of the user’s mood over the weeks that the app is being used. Every week for 4 weeks the user is asked how their mood is, and the user can control the expression of the face by a slider, from terrible to fantastic (Figure 6). Our motivation to include this feature is, partially, to collect data, bearing in mind the limitation that this is only an indication of the users’ moods and that personality is an influencing factor. However, there were other reasons to include mood charting in the app. Participants found this feature to be engaging, enjoyable, and empathic, which other studies have indicated may be particularly important for this target group [27,79]. Asking users how they feel is also a way to be polite and respectful. It was considered by users to be a good way to start before moving on to the intervention content. We believe that the mood charting functionality provided users with a sense of agency and that they appreciated being able to track their mood over time. Moreover, if the user does not want to chart their mood, they can skip this part, so there is also an offer of choice which may be important to feel a sense of autonomy in the UX [80-82].

#### Setting Goals

Taking action, even in small steps, is an important strategy to overcome avoidance behaviors and inactivity and because this is a challenge in the target population, we have introduced intention interventions into the RØST application. Within the chapters of RØST, participants will engage in the process of setting both long-term and short-term goals pertaining to work or education. To address common obstacles impeding goal achievements, such as distractions, detrimental habits, or aversion to necessary actions, we use concepts from mental contrasting (fantasizing about achieving the desired goal and reflecting on potential obstacles) along with implementation intention interventions (planning when, where, and how to take action toward the goal; MCII) [66-71]. Specifically, the users are asked to describe their goals. Then, they are asked to imagine how reaching that goal would feel, and what are the obstacles that prevent them from reaching the goal. The user is then asked to break down the goal into small steps, commit to a timeline where he or she will receive a reminder, and to set strategies to avoid potential obstacles. In the following module or week, the user is asked whether the goal was achieved or not. Then, the process is restarted with the definition of a new step and intention. This process of breaking down goals and setting reminders happens in every module during the use of the RØST application.

#### Audio and Video

An audio track was created to support the accessibility and user-friendliness of the app, recorded with professional actors. The experience from NSLM, U-SAY, and other studies of wise interventions was that using audio was a method to support flow and concentration, shutting out other noise [21]. By allowing audio consumption of the content, it is possible to use the app in a more relaxed manner, suitable on a mobile phone. From observing young adults on their phones, we noticed that they would often have their headphones on, so rather than listening to music or other things while consuming the contents of the app, we reasoned that using recorded audio tracks would allow for more concentration. Reading the intervention text would likely be faster for most readers, but going slow might mean a more careful reading and interpretation of content. The user can decide whether to use the app with or without audio by enabling or disabling the “listen” button, which is featured on top of every page.

In early testing, we showed a video to participants. This video summed up the growth-mindset concept in a simple manner. Participants could clearly explain the main messages of the video in their own words after watching the video and expressed that the use of video, even a simple one, is a more interesting way to consume the content compared with reading. In the final version of the app, we incorporated a simple animation video summing up the key message about growth mindset. Thus, it does not introduce any new concepts, but rather reinforces the messages. Incorporating video is a method to reach users who are not responding well to reading and helps reinforce learning through repetition.

#### Persuasive Design: Triggers, Praise, and Rewards

**Figure 7:**
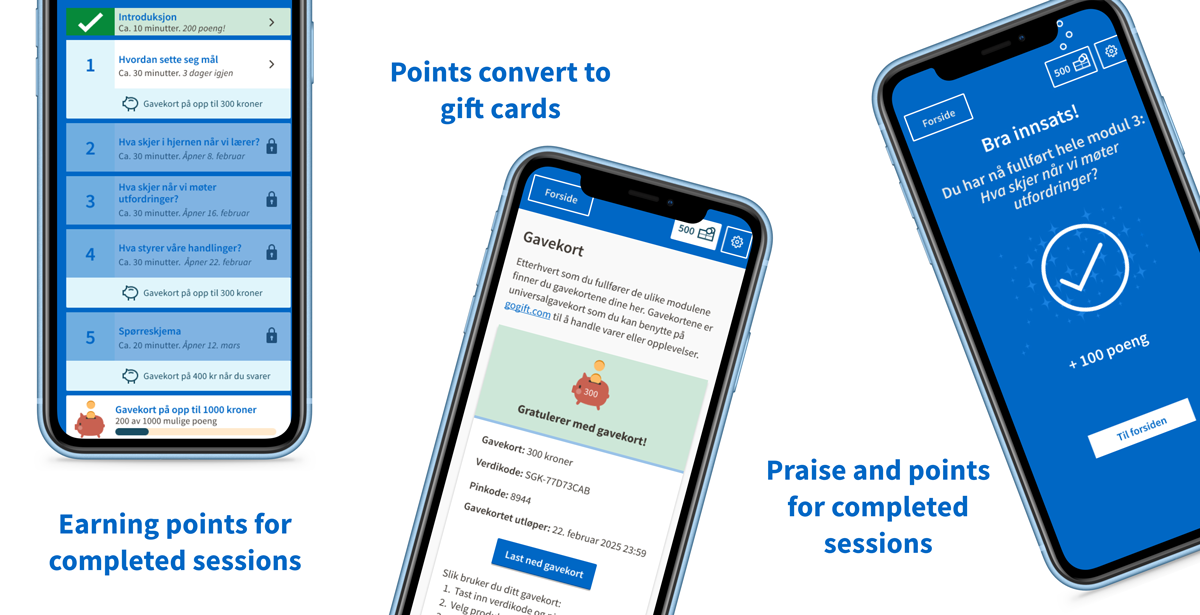
Rewarding adherence to the web app was implemented through points, gift cards, and praise. This was introduced into the application to overcome possible lack of intrinsic motivation to complete the modules of the intervention web application without the support of such features. The persuasive design features were adapted based on users’ feedback, the context, and the needs of the broader research project.

The participants who were involved in this study did not see any immediate gains for themselves from completing the intervention, even if they liked the app or found the messaging to be positive. On the basis of this lack of intrinsic motivation for use and review of past research on adherence to positive psychology apps, we introduced a set of “persuasive design features” to boost motivation and adherence, consisting of triggers (SMS text messaging notifications), praise, and rewards (points and gift cards). Figure 7 provides an overview of the persuasive design features.

The weekly interaction with the app is triggered by prompts in the form of SMS text messaging notifications and reminders. In a native or hybrid app, we could have sent push notifications to the users’ phones. However, we found it more cost-effective to build a web app. Another drawback of app notifications is their frequent dismissal or muting by users or their reluctance to grant app permissions. An alternative route was to choose emails or SMS text messaging. Our previous experience suggested that emails were not read by young adults in this user group but that SMS text messaging could be a good channel and therefore, this was selected as our trigger.

After receiving an SMS text messaging notification, the user has 1 week to complete the module. If the user has completed the content module in time, the user first receives *praise* in the app and then earns *points* which are converted to gift cards. The maximum earning for completing all content is roughly US $100 distributed over 3 gift cards.

If the module has not been completed by day 3, there are reminders sent out. If the user does not complete the module then it will expire at the end of week. The user can still view the module content but cannot earn any points or gift cards from completing the module. This is to create a sense of loss aversion [83] intended to nudge the user to complete the module within the set time frame. We expect that users who opt in to the study will complete or nearly complete the intervention to avoid missing out on their gift cards. We also hypothesize that if the user has invested time in completing the initial modules, this will reinforce the likelihood of them completing other modules as well [84].

Within the content modules, there are also progress bars and indicators to show how much of it is remaining. When the users can see that there is not much remaining and how much effort they have already invested in completing the session, they will also hopefully be less likely to drop out midway through the session, also building on the construct of loss aversion. At the top of every page, there is an indication of the points a user has earned. Points are counted as participants complete a session by animating the coins to land in a small wallet icon to the top right. This is also a form of visualization of what the user has achieved which may act as an additional pat on the back.

Theoretically, we expect these persuasive features of the app to assist in transcending the activation threshold [31] and increase the likelihood of adhering to the app throughout the necessary period.

## Discussion

### Principal Findings

This study used design methods to create an interactive intervention app based on positive psychologies targeting unemployed or inactive young adults. During an iterative process, with frequent usability testing with end users and input from stakeholders, the app evolved from concept sketches and early prototypes to a finished design ready for development. This paper presented the design and development of RØST, a web-based intervention app designed for mobile and desktop use. Through usability testing of prototypes, we could receive fast feedback and consider user insight in the next design iteration. Not only did the input from users lead to changes in design and functionality, but it also led to changes in content and its presentation. The intervention web app has been fully developed and is ready to be used on any device. With minimum adjustments, the web app may also be scaled up to be released to a broader population after being rigorously tested in planned forthcoming studies.

In this study, we have not only reported on the outcome of the study, which was the web app, but also detailed the process for how the end result came about. Rather than formalized analysis, we relied on action-oriented synthesis [44] and intuitive, abductive discovery [42]. In line with other design-oriented research, it is not always easy to see the direct link between the “research for design” [85,86], which was the feedback from end users and stakeholders, and the design itself [45]. The core team acted as interpreters and facilitators, balancing the opinions of the end users and stakeholders and insight into the context of unemployment in Norway, with past wise interventions, psychological theory, and theory on designing for behavior change and motivation.

A particular challenge to be addressed in the design and development of RØST was the adaption of content from existing interventions, designed for use in structured contexts, to this self-administered intervention app. This challenge was particularly important, given potential issues concerning motivation and structure in a user population of youth outside work and education. This challenge was addressed through a human-centered design process where end users and other stakeholders provided feedback on early prototypes that were iterated on several times. Through this process, we were able to develop more relatable examples and adapt to a new context, which is expected to be more useful for this population. To support the adaptation of existing intervention content, the development team established a set of principles and strategies for the human-centered design of RØST. Furthermore, we included features to ensure engagement and adherence via persuasive and interactive design elements. This research was thus informed both from the existing knowledge base of several evidence-based psychological and wise interventions and from end users and stakeholders involved throughout the development process.

**Figure 8:**
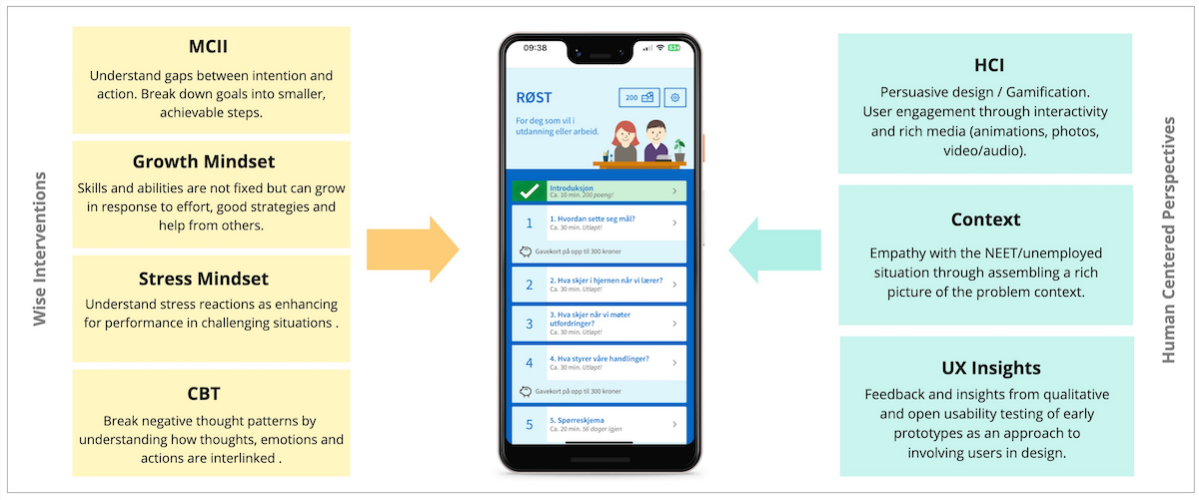
The RØST application is based on multiple sources and traditions of knowledge that are both theoretical and practice based. The core theoretical psychological underpinnings and strategies stem from various evidence-based wise interventions, and the user experience (UX) and adherence strategies stem from the human-centered design process and human-computer interaction (HCI) theory and evidence base. The resulting web application is a balancing act between these multiple perspectives and groundings. CBT: cognitive behavioral therapy; MCII: Mental Contrasting Intention Intervention; NEET: not in education, employment, or training.

Figure 8 depicts the dual grounding of the RØST application. On one hand it is based on a human-centered design process with participatory UX insights and theory from human-computer interaction, such as persuasive design, However, on the other hand, the app content and the psychologies it contains are developed by combining multiple evidence-based wise interventions.

Crafting effective digital interventions that are ready for use requires interdisciplinary work and skills within knowledge domains such as psychology, technology, and human-centered design [87]. Reflecting on the experiences of taking part in this research, we see that this may at times be a difficult balancing act. By elaborating on a real-world design research and development case in detail, we hope to contribute to a greater understanding of the complexity and many layers of decision-making and considerations that occur throughout such a process. Specifically, there is a potential for interactive technology bringing *more* to the interventions, moving beyond digitizing [87] and adapting past evidence-based interventions. Losing current restraints could lead to increased user engagement through new uses of interactive technology.

### Comparisons With Past Work: Designing for Use and Adherence

The user’s interests in the intervention content appeared to be minimal. This was seen not only in the evaluation of U-SAY and the early prototypes but also from our past interaction with the user group when exploring a gaming-based intervention [27]. Thus, a more visually pleasing and user-friendly app experience with relatable content will likely help reduce “friction” (lowering barriers to use). However, it will probably not be *sufficiently* *motivating* to get our target users to stick to the intervention from start to finish. Persuasive design [31] could be a way forward to boost motivation, as a lack of such features (eg, reminders, praise, and rewards) are potential shortcomings in web-based interventions [22]. Therefore, the completed RØST application includes praise, SMS text messaging triggers, and a scoring system leading to gift cards. The gift cards were pointed to by participants in our study as the biggest motivation factor for completing the program.

Though persuasive design may ensure adherence and get users to form and stick to new habits [88], there are also risks connected with the introduction of such features. They may undermine the intended therapeutic effects by adding more stress to the users [89]. This user group of unemployed young adults may be particularly sensitive to this kind of external pressure, even mild pressure like completing app content, if they already have depression or anxiety leading to avoidance. Furthermore, the provision of external incentives may also undermine intrinsic motivation [90]. The latter is not something we regard as particularly relevant because in this case, the baseline motivation is already low and as such, there is little risk of undermining intrinsic motivation via incentives when there is none to begin with [91]. We also consider the incentives provided in our study to be of a relatively low value so that it will not crowd out intrinsic motivation in those who are already motivated.

The use of persuasive design and gamification may be criticized as leading to engagement with a “flawed product,” as pointed out by Bogost [92]. Ideally, the intervention app should be motivating to use in itself. However, we consider this “superficial engagement” through gamification and persuasion to be ethically reasonable, if they contribute toward making the mundane fun while achieving changes that are beneficial to the users. This has been a successful approach within, for example, education, with the gamification of language learning apps such as Duolingo (Duolingo). However, the fine line between persuasion and “dark patterns” in UX design should be handled with great caution, and further research is needed to evaluate whether and how these persuasive and gamification design features impact the effectiveness of the app and its user motivation.

### Strengths and Limitations

There were a number of needs and considerations related to forthcoming experimental studies (RCTs) that shaped the design process in this study. The strengths of this approach to development are that the app will be tested for effectiveness in large-scale experiments and is built on evidence-based intervention designs. A weakness of this approach is that controlling variance influences and shapes the UX design in a way that could itself diminish engagement and user satisfaction.

Other weaknesses relate predominately to our methodology of capturing user feedback on prototype experiences. Thus, the user feedback and observation of app use were not based on ready-to-use solutions but on a range of prototypes. The method was chosen specifically to be able to take participant contribution and input into account in the designed outcome, but limits to some degree the reliability of the insights reported. There were also a limited set of participants recruited, and they all received rewards to contribute to the usability testing. We cannot know whether the users we engaged in the study were more prone to see the importance of rewards since they were already being rewarded to take part. Furthermore, most of the prototypes included some form of reward scheme or gamification built-in, either points, lottery, praise, money, or social features. Being aware of this limitation, we have built 2 versions of the app, one version which includes the gift cards and points and another one where we have removed this feature. Both versions of the app were tested and compared in a recent study (not yet published).

Another limitation is the lost opportunity of reporting more specifically on the stakeholder perspectives. In this study, stakeholders were not recruited as research participants, and their opinions or influence on the intervention app were not seen as central to the research question. However, in retrospect, perhaps a more complete documentation of the process, including stakeholder opinions could have contributed to enriching the picture of the research.

Furthermore, we do not venture into a discussion on whether the psychologies and content used in the app are indeed appropriate for the target group of unemployed young adults and for the desired outcome set for the intervention. For example, the MCII intervention, especially the goal-setting aspect, may not be suitable for users who lack specific objectives or set unrealistic goals, potentially leading to stress. Whether or not the selected positive psychologies used are appropriate for the target population will be explored further during larger scale experimental studies to understand its effects.

### Conclusions

Involving end users in the development of a self-administered wise intervention enabled relatable content development and resolved potential usability problems in the adaptation of several evidence-based interventions into a web app specifically designed for a population of unemployed young adults. This approach also confirmed the need for increasing user motivation through supportive persuasive functionality, such as notifications, reminders, praise, and rewards. Such persuasive features are likely pivotal for promoting signup and adherence to the intervention. The results of this study have several implications. An essential implication is to enact and cater to end-user feedback and observation through an application-oriented developmental protocol extended by user insights, as well as to be guided by psychologically informed design. Furthermore, we identified a challenge in harmonizing the flexible interactivity that users wanted and needed to feel engaged with an evidence-based research approach. The lack of interactivity and catering for responsive user journeys identified may contribute to a suboptimal UX. Future research should apply design methods to these issues to resolve them and explore whether it is possible to include user perspectives not only in the refinement and development of a solution to make it self-administered but also in the identification of and definition of the problem that the intervention is trying to tackle. Nevertheless, we conclude that the RØST application is ready to be tested and used in further research with users to measure intervention effectiveness and to get a greater understanding of use, reach and adherence, and the effect of incentives introduced in such an app. Although the app is designed specifically with research needs in mind, it may be implemented relatively easily as a ready-to-use app outside of the research domain.

## Appendix

Multimedia Appendix 1 Description of the RØST app.

## Abbreviations

MCII: Mental Contrasting Intention Intervention
NAV: Norwegian Labour and Welfare Administration
NSLM: National Study of Learning Mindsets
RCT: randomized controlled trial
UI: user interface
UX: user experience

## Data Availability

The datasets generated during and analyzed during this study are available from the corresponding author on reasonable request.
