# Supplementary material for: A Web-Based Intervention to Support a Growth Mindset and Well-Being in Unemployed Young Adults: Development Study
Source: JMIR Form Res. 2024 Nov 8;8:e59158. doi: 10.2196/59158 (PMC11584549; doi:10.2196/59158)
Supplement: Multimedia Appendix 1 [file formative_v8i1e59158_app1.pdf]

A Web-Based Intervention to Support Growth Mindset and Well-Being in Unemployed Young Adults: Development Study

Multimedia appendix

Table 1. RØST intervention components

<table>
<tr><th>Module</th><th>Content</th></tr>
<tr><td>

0. Introduction

</td><td>

- What other young people are saying about the program (establish trust, relevancy and norms)
- Two real-world examples about other young people who have struggled but changed how they think and act
- We need your help and story to help others (empathy)
- Introduce program core content (growth mindset)
- Overview of complete program expectation)
- Overview of next module

</td></tr>
<tr><td>

Module 1: How to set goals?

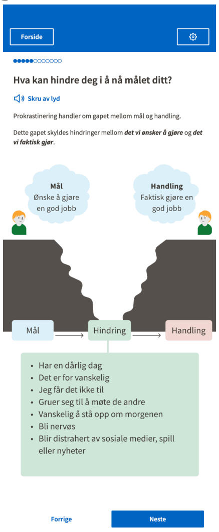

</td><td>

- How do you feel? (mood charting)
- Introduce procrastination and gap between thinking and doing
- Teach importance of goal setting and breaking down task into smaller micro-goals
- User sets a goal, describes possible positive outcomes if the goal is reached and how that would feel.
- User sets "next little step" and commits to a time and a day to receive an SMS reminder to do the next step.
- Foresee possible obstacles and strategies to overcome them (such as "If I'm distracted by my phone i will put it in airport mode)
- Asked to rate the app
- Summing up core ideas
- Overview of next module
- Asked about time of day user wants to receive the SMS reminders (morning or evening)
- Links to resources from NAV such as "how to write a CV"
- Praise and scores

</td></tr>
<tr><td>

Module 2: What happens in the brain when we are learning?

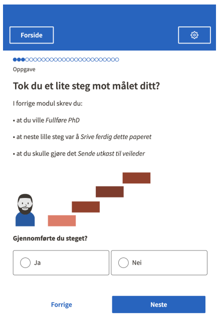

</td><td>

- How do you feel? (mood charting)
- Repeat previous session content about the gap between goals and actions
- Follow up on the set goal and "next little step" (repeat what user had written as their goal and next little step for reaching this goal).
- Theory: Latest news about the brain, including statements from other young people who have been through the program
- Task: Describe a demanding task, something you struggled with.
- Provide two relatable examples, lived experiences
- Quotes from young people.
- Task: Have you ever been told you were not good at something?
- Theory: You can train your brain + introduce the metaphor that the brain is like a muscle.
- Task: Tell about a time when you worked hard and may have developed stronger connections in your brain.
- Play video: How do we know that the brain can be stronger
- Statement from researcher at Stanford supporting the message
- Introduce that it's not just about efforts, but about trying different strategies
- Task: Can you tell us about a time where you solved a task using one of these strategies
- Theory: Develop the brain by learning from mistakes
- Task: Help a friend who has given up

</td></tr>
</table>

<table>
<tr><td></td><td>

- Learning from successful people: Natural talent vs hard work (choose to read about Christiano Ronaldo, John Legend, J.K. Rowling or founders of Apple)
- Focus on your own learning: Focus on what can you do to develop yourself
- Set next step in order to reach your goal (the goal set in module 1)
- Rate Rost app (star rating)
- Next week
- Praise and scores

</td></tr>
<tr><td>

Module 3: What happens when we face challenges?

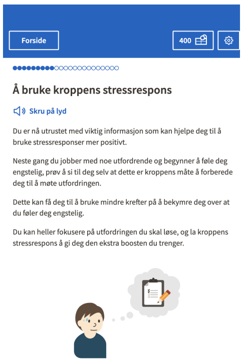

</td><td>

- How do you feel? (mood charting)
- Recall from last session: Gap between objectives and action
- Did you take the small step? (follow up on goal set in last session)
- Reflection task: Recall a difficult task
- Psycho-education: Unpleasant feelings may be useful (explain and normalize stress reaction)
- Strategies for overcoming stressful situations
- Relatable examples: Other young people worrying about exam and job interview
- Some stress can also be negative - seek help (contact information to health professionals)
- Help us explain this to other young people by sharing your own experience
- Set new goal including time and date
- Overview of next module
- Praise and scores

</td></tr>
<tr><td>

Module 4: What guides our actions?

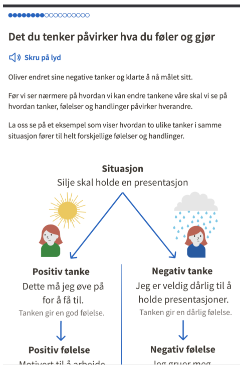

</td><td>

- How do you feel? (mood charting)
- Psychoeducation: Feelings may be changed
- Did you take the small step? (follow up on goal set in last session)
- Case example: Oliver, who learnt how to change his thinking
- Psychoeducation: What you think influences your actions
- Psychoeducation: Understanding negative thought patterns
- Psychoeducation: How to break negative thought patterns
- The power of "not yet"
- Case study: IT-company who employs unemployed gamers
- Repeating previous learning on growth mindset
- Task: Choose strategies and reflect on your choice
- If you need further help (reference to health professionals)

</td></tr>
<tr><td>

Module 5: Questionnaire

</td><td>

This session does not include learning content, but is set up to direct users to the survey tool for the final measures. The final gift card is awarded upon completing this session.

- Thank you for completing ROST
- Take surveys
- Receive gift card

</td></tr>
<tr><td>

Placebo

</td><td>

The design also included a placebo-version of the app, which has a similar user experience and user interface design, but does not include the intervention content, only generic job-seeking advice. Useful information, but nothing different compared to what users receive from the public services already.

</td></tr>
<tr><td>

Non-reward version

</td><td>

We have developed a version of the app which includes praise and reminders but does not have any points or rewards (giftcards). This was developed in order to understand better how users adhere to the intervention in a non-reward setting.

</td></tr>
</table>

Figures excluded from the paper manuscript

We have included here in this appendix some figures that had to be excluded from the manuscript due to length. However, we have incorporated them here in order to ensure the best possible transparency of this development study.

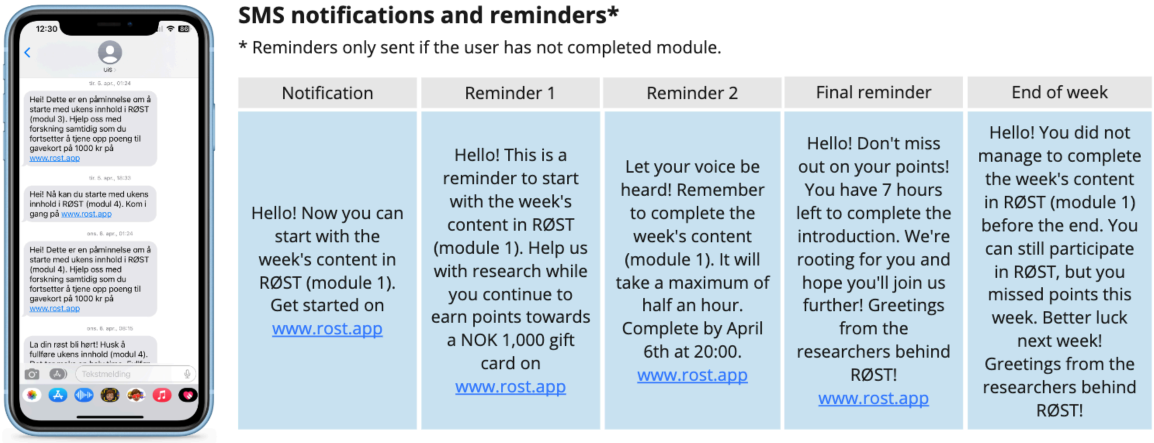

Figure 1. SMS notifications and reminders (here translated to English) are sent out to trigger interaction with the web-app and to support adherence. SMS was chosen specifically to ensure delivery, compared to in-app notifications or email.

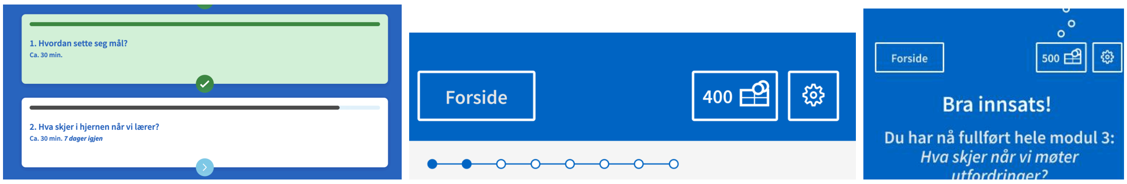

Figure 2. Progress bars and module completion and keeping track of points earned visualizes achievement, earnings and progress, building on psychological concepts such as loss aversion and set completion.

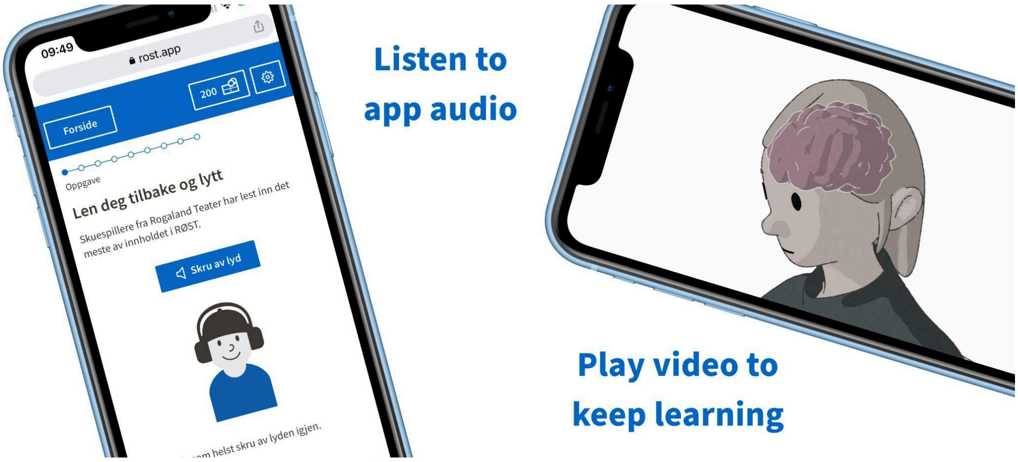

Figure 3. Audio and video was implemented into the web-app in order to increase accessibility and user friendliness.

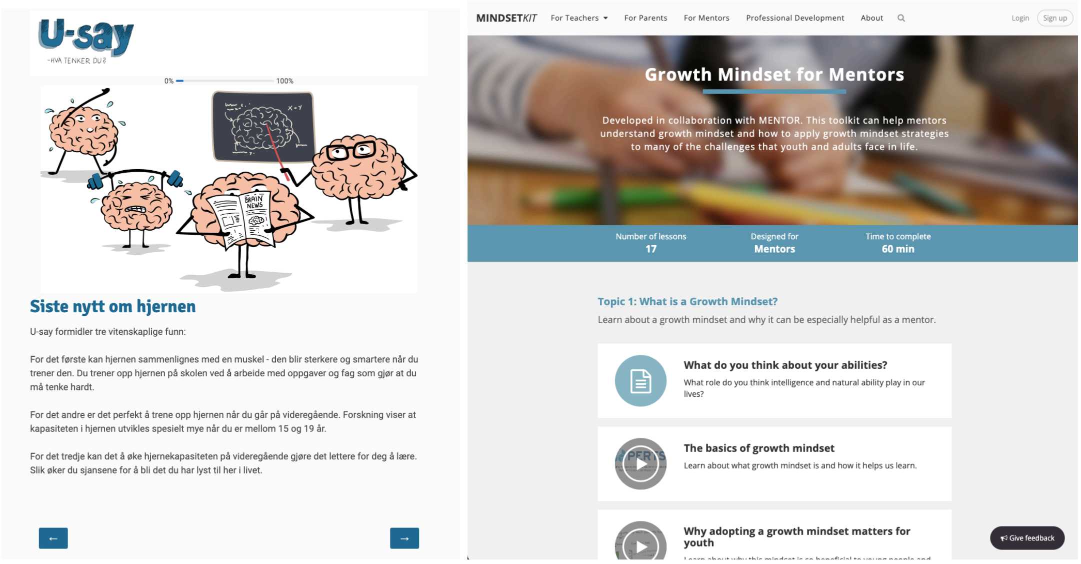

Figure 4. Screenshots of the U-SAY intervention (left) contrasted against PERTS Mindset Kit for Mentors (right), demonstrating the different styles of communication in tone and visual design and user experience.

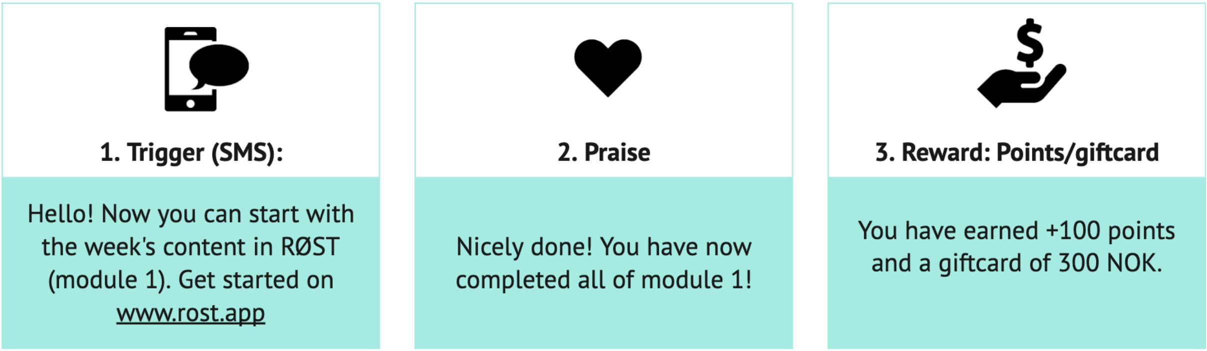

Figure 5. Example of the flow of triggers, praise and reward in the RØST-app.
